# Annotation of pseudogenic gene segments by massively parallel sequencing of rearranged lymphocyte receptor loci

**DOI:** 10.1186/s13073-015-0238-z

**Published:** 2015-11-23

**Authors:** Jared Dean, Ryan O. Emerson, Marissa Vignali, Anna M. Sherwood, Mark J. Rieder, Christopher S. Carlson, Harlan S. Robins

**Affiliations:** Adaptive Biotechnologies, Seattle, WA USA; Fred Hutchinson Cancer Research Center, Seattle, WA USA

## Abstract

**Background:**

The adaptive immune system generates a remarkable range of antigen-specific T-cell receptors (TCRs), allowing the recognition of a diverse set of antigens. Most of this diversity is encoded in the complementarity determining region 3 (CDR3) of the β chain of the αβ TCR, which is generated by somatic recombination of noncontiguous variable (V), diversity (D), and joining (J) gene segments. Deletion and non-templated insertion of nucleotides at the D-J and V-DJ junctions further increases diversity. Many of these gene segments are annotated as non-functional owing to defects in their primary sequence, the absence of motifs necessary for rearrangement, or chromosomal locations outside the TCR locus.

**Methods:**

We sought to utilize a novel method, based on high-throughput sequencing of rearranged TCR genes in a large cohort of individuals, to evaluate the use of functional and non-functional alleles. We amplified and sequenced genomic DNA from the peripheral blood of 587 healthy volunteers using a multiplexed polymerase chain reaction assay that targets the variable region of the rearranged TCRβ locus, and we determined the presence and the proportion of productive rearrangements for each TCRβ V gene segment in each individual. We then used this information to annotate the functional status of TCRβ V gene segments in this cohort.

**Results:**

For most TCRβ V gene segments, our method agrees with previously reported functional annotations. However, we identified novel non-functional alleles for several gene segments, some of which were used exclusively in our cohort to the detriment of reported functional alleles. We also saw that some gene segments reported to have both functional and non-functional alleles consistently behaved in our cohort as either functional or non-functional, suggesting that some reported alleles were not present in the population studied.

**Conclusions:**

In this proof-of-principle study, we used high-throughput sequencing of the TCRβ locus of a large cohort of healthy volunteers to evaluate the use of functional and non-functional alleles of individual TCRβ V gene segments. With some modifications, our method has the potential to be extended to gene segments in the α, γ, and δ TCR loci, as well as the genes encoding for B-cell receptor chains.

**Electronic supplementary material:**

The online version of this article (doi:10.1186/s13073-015-0238-z) contains supplementary material, which is available to authorized users.

## Background

During T-cell development, immature T-cells undergo somatic rearrangement of their T-cell receptor (TCR) loci within the thymus [1]. This rearrangement accounts for the vast sequence diversity found in mature TCRs, which in turn allows TCRs to bind to the great diversity of antigens presented by major histocompatibility complex molecules on the surface of other cells. The TCR protein is composed of two molecules, encoded by the TCRα and the TCRβ genes (or, in a small proportion of T-cells, by the TCRγ and δ genes). Diversity in the TCRβ and TCRδ chains results from the recombination of a large number of variable (V), diversity (D), and joining (J) gene segments, whereas only V and J gene segments recombine to generate the TCRα and TCRγ chains. Additional diversity is achieved by deletion and non-templated insertion of nucleotides at the junctions [2]. A similar process occurs in B-cells, and results in the generation of heavy and light chains of the immunoglobulin receptors and secreted antibodies.

Different sources report variable numbers of V, D, and J gene segments for the TCRB locus [2–4]; for example, the international ImMunoGeneTics (IMGT) database reports 68 TRV, 14 TRJ, and 2 TRD gene segments, corresponding to 146, 16, and 3 alleles, respectively, including both functional and non-functional alleles [4]. However, it is likely that there is some variation in gene numbers within the human population. Recent studies indicate that true polymorphisms are easily missed because of limitations in the number and diversity of individuals analyzed through low-throughput methods [5, 6], both in ethnically diverse populations such as Papua New Guineans and Mexicans, and in well-studied Caucasian cohorts (reviewed in [7]). The same sequencing studies have suggested that many polymorphisms have been reported in error [5]. Thus, we sought to evaluate the presence of functional and non-functional alleles in a large cohort of healthy individuals using high-throughput sequencing of the variable region of the beta chain of the TCR.

It is estimated that several million distinct TCRβ sequences (measured by counting unique rearranged complementarity determining region 3 [CDR3] sequences) are present in the peripheral blood of a typical human, including many TCRβ sequences that utilize each of the available V, D, and J gene segments [8]. The repertoire of germline gene segments comprising the genomic TCRβ locus is therefore an important contributor to sequence diversity in naïve T cells, and thus to the ability of the adaptive immune system to engage pathogens and to recognize aberrant proteins such as those generated by tumor cells [9].

Owing to the random nature of sequence editing during VDJ recombination, most rearrangements result in non-functional TCRβ genes: either a stop codon is created, the V and J gene segments are not in the same coding frame, or a pseudogenic gene segment that encodes a major defect outside the CDR3 region is incorporated. T cells in which the first VDJ rearrangement results in a non-functional TCRβ gene often undergo VDJ recombination of their second allele, thus allowing the cell a second chance to rearrange a functional TCRβ [10]. After gene rearrangement, positive selection in the thymus ensures that all mature T cells have at least one TCRβ allele encoding a functional TCRβ protein; T cells carrying only non-productive rearrangements undergo apoptosis [11]. In addition, some pseudogeneic alleles carry non-functional Recombination Signal Sequences (RSSs), which prevent their incorporation into rearranged genes [12, 13].

By definition, pseudogenes cannot encode for productively rearranged receptor genes, and therefore can only be observed as the second allele in cells that also express a functional TCRβ [11, 14]. Functional gene segments can be observed both in productive and in non-productive rearrangements. Herein, we take advantage of this fact to evaluate the use of functional and non-functional alleles of V gene segments in a large cohort of healthy volunteers. Because pseudogenic gene segments can only be observed in non-productive rearrangements, there is no selective pressure to ensure that CDR3 length is a multiple of three or that it lacks a stop codon, and thus rearrangements at the TCRβ locus including these gene segments should lead to genes with key motifs that are in-frame and have no premature stops less than one third of the time. In contrast, functional genes will have a much higher rate of CDR3 sequences that are in frame with the flanking V and J genes, because they must be in-frame and free of stop codons in all cases where they are part of a productive rearrangement, in addition to being in-frame one third of the time they are part of a non-productive rearrangement. For example, if we assume that 80 % of rearrangements of functional genes are productive, functional genes would be in frame approximately 87 % of the time (i.e., 80 % plus one third of 20 %).

Multiplex amplification across the VDJ junction allows direct sequencing of the genomic DNA of TCRβ CDR3 regions from millions of T cells simultaneously [8], thus enabling a much deeper analysis than previously possible. By sequencing tens of millions of TCRβ CDR3 sequences from hundreds of healthy volunteers and analyzing the frequency of unique productive and non-productive rearrangements that contain each TCRβ V gene segment, we derived rules that allow the classification of individual immune gene segments into functional and non-functional categories. Comparison of these results to IMGT [15], the most widely used immune gene database, showed that our data agree with the current functional annotation of the majority of TCRβ V gene segments. However, our results suggest that two TCRβ V gene segments that were only reported to contain functional alleles were overwhelmingly seen as non-functional alleles in our cohort, suggesting the existence of novel pseudogenic alleles that are present at high frequency in this population. We also identified a subset of TCRβ V gene segments that had not previously been recognized as having multiple alleles, whose functional status segregated in our cohort. Finally, two genes currently annotated as having both functional and non-functional alleles behaved uniformly in our cohort, one as a functional gene and the other as a pseudogene.

## Methods

### Experimental cohort

Human peripheral blood samples were obtained from healthy volunteers under a protocol following written informed consent approved and supervised by a Fred Hutchinson Cancer Research Center Institutional Review Board. The research included in this work conforms to the Helsinki Declaration. Further details of the cohort are given in [16]; data from 587 individuals in that cohort (i.e., those with data available at the time of writing this study) were used in the secondary analysis included in this work. The sequencing data for the 587 individuals can be accessed from www.adaptivebiotech.com/pub/Dean, and has also been deposited in the Dryad Digital Repository (doi:10.5061/dryad.t47g3).

### High-throughput TCRβ sequencing

Genomic DNA was extracted from cell samples using the Qiagen DNeasy Blood Extraction Kit (Qiagen, Gaithersburg, MD, USA). We amplified and sequenced the CDR3 region of the rearranged TCRβ genes using previously described protocols [8, 17]. Briefly, a multiplexed polymerase chain reaction (PCR) method was employed using a mixture of 60 forward primers specific to TCR Vβ gene segments and 13 reverse primers specific to TCR Jβ gene segments, and 87 base pair (bp) reads were obtained using the Illumina HiSeq System (Illumina Inc., San Diego, CA). Raw HiSeq sequence data were preprocessed to remove errors in the primary sequence of each read, and to compress the data. A nearest neighbor algorithm was used to collapse the data into unique sequences by merging closely related sequences, to remove both PCR and sequencing errors. The TCRβ CDR3 region, as defined by the IMGT collaboration [18], begins with the second conserved cysteine encoded by the 3′ portion of the V gene segment and ends with the conserved phenylalanine encoded by the 5′ portion of the J gene segment. The number of nucleotides between these codons determines the length and therefore the frame of the CDR3 region. Each sequence was required to have a minimum six-nucleotide match to one of the V gene segments and one of the J gene segments. To ensure that our conclusions were conservative, sequences that did not unambiguously match a single V gene segment (because of deleted nucleotides or errors) were excluded from our analyses. This resulted in the exclusion of data from five V gene segments annotated as functional in the IMGT database because they did not contain any unique nucleotide sequence within our 87-bp sequencing reads (TRBV6-2 and TRBV6-3). Next, each rearrangement was scored as productive if (a) there were no stop codons in the reading frame of the CDR3 region, and (b) it was in frame with the V and the J gene (i.e., CDR3 length was a multiple of three nucleotides); or classified as non-productive otherwise.

### Data analysis

We calculated the proportion of time that each TCRβ V gene segment was identified as being part of a productive rearrangement, the average of this metric for each gene segment in the cohort, and its standard deviation among all individuals. To define a threshold for functional and non-functional (i.e., pseudogene/open reading frame [ORFs]) genes, we first assumed that the current annotations present in the IMGT database are mostly correct, and thus calculated the median proportion of productive rearrangements both for gene segments currently annotated as functional and those currently annotated as non-functional; the median was used to avoid statistical artifacts from a small number of gene segments whose annotations might not match the alleles present in our cohort. Also, gene segments with known functional and non-functional alleles were ignored for this calculation. In an attempt to re-classify gene segments based on these medians, we used the midpoint to define 56.7 % as the threshold separating functional from non-functional genes.

To identify genes with both functional and non-functional alleles segregating within our cohort, we looked for substantial differences in the proportion of productive rearrangement between individuals. Specifically, we segregated gene segments into “fixed” (consistently functional or consistently non-functional) and “segregating” (functional and non-functional alleles) classes. To capture gene segments with concordant versus discrepant behavior across individuals, we used the standard deviation of the proportion of productive rearrangements as a metric. Again assuming that most IMGT annotations are correct but there are some outliers, we defined the median of each group using current annotations and took the midpoint of those medians as a threshold. This midpoint was then used to identify a threshold value of standard deviation (5 %) to annotate gene segments as either fixed or segregating with respect to functionality.

Final annotation for each gene segment was performed as follows: gene segments that displayed a standard deviation above 5 % were deemed to have both types of alleles (i.e., functional alleles and non-functional pseudogene/ORF alleles) segregating in our cohort. For gene segments with a standard deviation below 5 %, we annotated them as functional if they were seen in productive rearrangements 56.7 % of the time or more, and as non-functional/pseudogenes otherwise.

## Results and discussion

In order to functionally annotate human TCRβ V gene segments, we performed deep sequencing of the TCRβ CDR3 regions on a set of PBMC samples obtained from 587 healthy volunteers [16]. Genomic DNA was obtained from these samples, amplified using a multiplex PCR assay that targets the rearranged TCRβ CDR3 region, and sequenced as previously described [17]. Each CDR3 sequence was aligned against reference sequences in the IMGT database to determine which V, D, and J gene segments were utilized in each rearrangement [15]. Sequences were deemed to be productive if the rearranged gene segments were observed to maintain the coding frame between the V gene segment, the CDR3 region, and the downstream J gene segment, and no premature stop codons were detected. In total we generated 276 Gb of sequence data, and observed 117 million unique TCR sequences with unambiguous V gene classification from an estimated total of 179 million total T cells profiled. The data can be accessed from www.adaptivebiotech.com/pub/Dean.

**Fig. 1:**
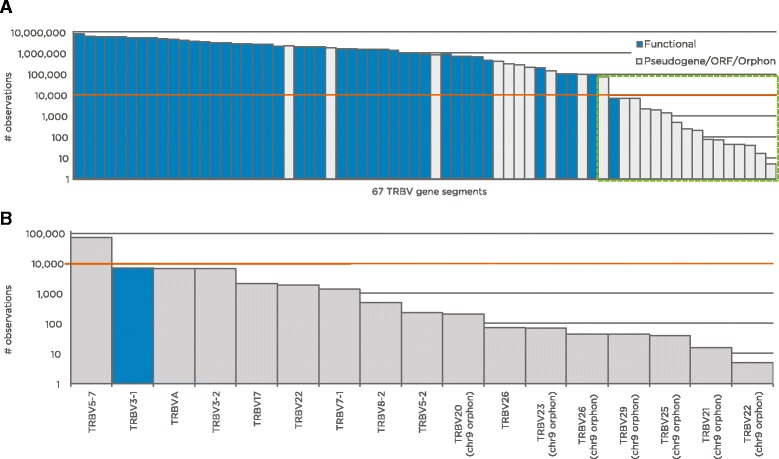
Abundance of each TCRβ V gene segment in our study. **a** The number of TCRβ gene rearrangements that aligned to each of 67 V gene segments, are ordered by abundance. Values varied widely, from 9 million matches to TRBV5-1 to five matches to TRBV22 (chromosome 9 orphon). The *colors* indicate the current IMGT annotation (*blue*: functional or mixed; *gray*: pseudogene or open reading frame (*ORF*). A large discontinuity in abundance occurs between TRBV5-7 and TRBV3-1; panel **b** magnifies this region. The *red line* in both panels indicates the threshold for each gene segment to be included in further analyses

First, we needed to identify gene segments with sufficient representation in the data so that we could meaningfully assess the ratio of productive to non-productive rearrangements; we also assessed overall representation for each gene segment because gene segments with very low representation in our data are likely to have non-functional RSS sequences and be classified as pseudogenes on that basis alone [12]. Figure 1 displays the number of total rearrangements aligning to each of 67 V gene segments (including several orphons from chromosome 9 as negative controls). There was substantial discontinuity of approximately 10-fold between TRBV5-7 (observed 74,000 times for a mean of 125 rearrangements per individual) and TRBV3-1 (observed 7100 times for a mean of 12 rearrangements per individual). All gene segments less frequent than TRBV5-7 (with the exception of TRBV3-1 itself) are currently annotated as pseudogenes, and all chromosome 9 orphon gene segments are in this group (we presume that the alignment to orphons is due to fortuitous PCR or sequencing errors derived from their paralogs on chromosome 7). Because of the considerable discontinuity, genes below this level are overwhelmingly pseudogenic according to current annotations, and because there would be a large amount of noise in estimating the proportion of productive rearrangements given a mean of 12 rearrangements per individual, the 17 gene segments (including orphons) less frequent than TRBV5-7 were excluded from any downstream analyses. In total, this process left 51 TRBV gene segments for analysis, among which there was a significant relationship between pseudogenic annotation in IMGT and less-frequent gene segment utilization in our cohort (*p* = 0.001 by two-tailed Mann–Whitney U test), perhaps indicating a lack of selection for an efficient RSS among pseudogenic gene segments.

**Fig. 2:**
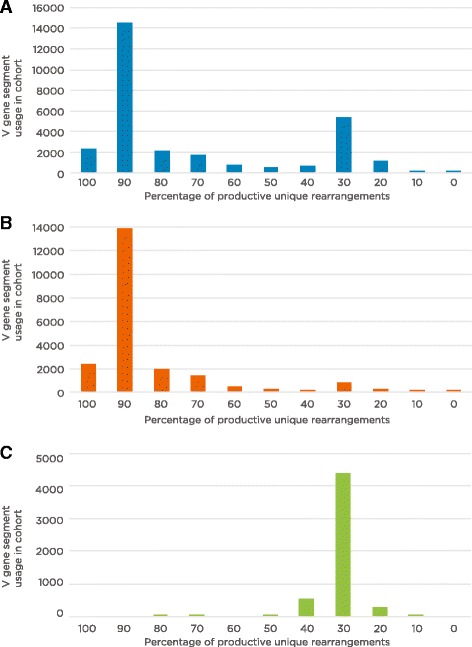
Frequency of productive rearrangement percentages for TCRβ V gene segments. For each individual, we calculated the percentage of productive unique rearrangements for each V gene segment found in the sample. The values were then binned in increments of 10 % (e.g., those with values between 0 and 10 % are included in the ‘10’ bin), with the height of the bars representing the number of times each percentage range was found in the cohort. **a** Frequency of productive rearrangement percentages for all 51 TCRβ V gene segments used in this study. The majority of V gene segments are found productively rearranged either ~90 % or ~30 % of the time. **b** Frequency of productive rearrangement percentages for the 37 TCRβ V gene segments used in this study that are annotated as functional in the IMGT database. The majority of these gene segments are found within productive rearrangements of the TCRβ gene ~90 % of the time. **c** Frequency of productive rearrangement percentages for the nine TCRβ V gene segments used in this study among V gene segments annotated as pseudogenes or open reading frames in the IMGT database. The majority of these gene segments are found within productive rearrangements of the TCRβ gene ~30 % of the time

For each of the 51 TCRβ V gene segments that had sufficient representation and unambiguous V gene segment assignment in our dataset (Additional file 1), we calculated the mean percentage of productive rearrangements in each individual. The resulting distribution shows that, at the population level, most TCRβ V gene segments were found in productive rearrangements either ~30 % or ~90 % of the time (Fig. 2a), suggesting they are either non-functional or functional, respectively. When only TCRβ V gene segments that are annotated as functional on the IMGT database were considered in this analysis, the majority of rearranged TCRβ V gene segments were found in productive rearrangements ~90 % of the time (Fig. 2b). Likewise, TCRβ V gene segments annotated as either pseudogenes or ORFs on the IMGT database were generally found in productive rearrangements ~30 % of the time (Fig. 2c).

**Fig. 3:**
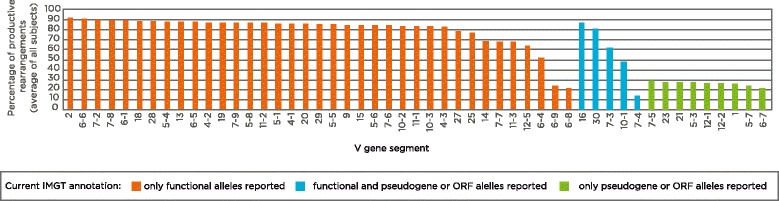
Percentage of productive rearrangements for individual TCRβ V gene segments. Shown are all TCRβ V gene segments included in this study, classified based on their current IMGT annotation as either functional gene segments (*orange*), having multiple alleles of differing functionality (*teal*), or pseudogenes/open reading frames (*ORFs*; *green*), sorted by the mean percentage of productive rearrangements within each category. The majority of functional gene segments are found in productive rearrangements most of the time (median 85.5 %). Gene segments with both functional and non-functional alleles display a wide range of productive rearrangements between 15 and 85 %, whereas all gene segments currently annotated as either pseudogenes or ORFs are found in productive rearrangements less than 30 % of the time (median 26.9 %)

The data for each of the 51 TCRβ V gene segments included in our dataset are shown in Fig. 3. The majority (34 out of 37) of TCRβ V gene segments annotated in IMGT as functional displayed a mean percentage productive rearrangement above 56.7 %, and thus our data are consistent with their current annotation [15]. However, TCRβ V gene segments TRBV6-8 and TRBV6-9, for which only functional alleles are currently reported in the IMGT database, were found in productive rearrangements only 21.8 % and 23.7 % of the time, respectively, suggesting that only non-functional alleles for these genes were observed in our cohort. In contrast, the five V gene segments in our dataset that have some functional alleles and some pseudogenes or ORFs, as per IMGT, displayed percentage productive rearrangements that ranged between 14 and 86 %. This observation suggests that the frequency of the functional versus the non-functional alleles for these V gene segments varies widely. It is also possible that some individuals in our cohort are heterozygote and carry both a functional and a non-functional allele for these segments. Finally, all nine TCRβ V gene segments annotated as either pseudogenes or ORFs on the IMGT database for which we had sufficient data were found in productive rearrangements at frequencies lower than 56.7 %, in agreement with their current annotation as non-functional gene segments [15].

**Fig. 4:**
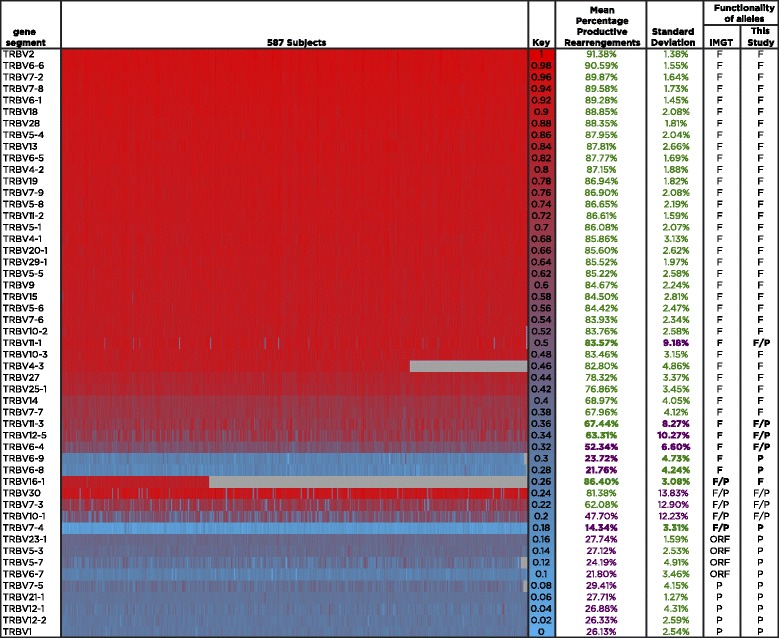
Heat map showing the percent productive rearrangements for all TCRβ V gene segments throughout the cohort. The percentage of productive TCRβ V rearrangements found in mature T cells is shown for each of the 587 individuals in the cohort for each V gene segment included in this study (listed to the left of the heat map). The column labeled “Key” indicates the color code: gene segments found in a high percentage of productive rearrangements in that individual are indicated in *red*, while gene segments with low frequencies of productive rearrangement in that individual are shown in *blue*. The next two columns indicate the mean percentage of each V gene segment found in productive rearrangements across all individuals; values over 56.7 % are displayed in *green* while values under 56.7 % are shown in *purple*. The next column corresponds to the standard deviation, with values under and over 5 % shown in *green* and *purple*, respectively. V gene segments are classified by their current IMGT annotation as shown in the column labeled IMTG (*F* functional, *P* pseudogene, *ORF* open reading frame, *F/P* or *F/ORF* genes with multiple alleles that differ in their functional annotation), and sorted by percent productive rearrangement within each category. The last column indicates the functionality of the alleles observed in this study. Discrepancies annotations between this study and IMGT are bolded

Figure 4 shows the percent productive rearrangement data for all TCRβ V gene segments in every individual in the cohort using a heat map. As before, our analysis agreed with the IMGT annotations for the majority of the TCRβ V gene segments currently annotated as functional, as well as those currently annotated as pseudogenes or ORFs, in particular for all those with standard deviations below 5 %. This analysis also confirmed that TRBV6-8 and TRBV6-9, both of which have consistently low values in all individuals, were only seen as non-functional alleles in our cohort. In addition, it is clear that gene segments with high standard deviations appear productive in some individuals and pseudogenic in others. In agreement with this, three of the genes with the highest standard deviation in our study (TRBV30, TRBV7-3, and TRBV10-1) are annotated on the IMGT database as having both functional and non-functional alleles, suggesting that they can rearrange to produce functional TCRs in some individuals and not in others. In addition to this, our data indicate that TRBV11-1, TRBV11-3, TRBV12-5, and TRBV6-4, all currently annotated as functional genes but having standard deviations above the 5 % threshold, likely have non-functional alleles that have not been described to date.

Among the four gene segments with a large representation of functional and non-functional behavior in our cohort (i.e., those displaying a proportion of productive rearrangements above the functional threshold in 10–90 % of individuals: TRBV6-4, TRBV7-3, TRBV10-1, and TRBV12-5), a small but significant correlation was observed between the functional/pseudogene status of the four genes, suggesting the possibility of linkage disequilibrium between alleles of the various TCRβ V gene segments (Table 1). Alternatively, it is possible that some unknown experimental factor could cause these gene segments to appear functional or non-functional in the sequencing data from each individual.

**Table 1:**
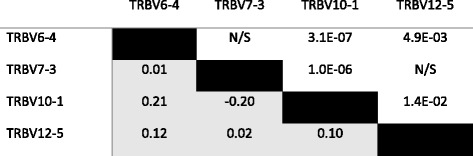
Correlation of functional/pseudogene status among gene segments

Correlation of functional status among individuals for the four TCRβ V gene segments that were found to be functional in between 10 and 90 % of our experimental cohort. Lower left of the table, below black cells: Pearson’s r (calculated on a binary classification of each gene segment as functional or pseudogene in each individual, N = 587). Upper right of the table, above black cells: *P*-values matching each correlation coefficient, calculated by normal approximation

Finally, although only observed in 186 individuals in our cohort, TRBV16-1, which is annotated in IMGT as a gene segment with functional and non-functional alleles, behaved in our cohort as a fully functional gene segment, displaying a percentage productive rearrangement of 86.4 % and a standard deviation of 3 %, whereas TRBV7-4, also currently annotated as having both functional and non-functional alleles, actually had the lowest percentage of productive rearrangements of all gene segments analyzed and a low standard deviation, and thus consistently behaved as a pseudogene in our cohort.

## Conclusions

By assessing high-throughput sequencing data derived from peripheral T cells that have undergone thymic selection in a large cohort of healthy individuals, we annotated TCRβ V gene segments as either functional, pseudogene/ORFs, or as having both functional and non-functional alleles. We found that most TCRβ V gene segments currently annotated as functional genes in the IMGT database were observed as in-frame rearrangements approximately 90 % of the time, whereas the vast majority of TCRβ V gene segments annotated as either pseudogenes or ORFs by IMGT were found as in-frame rearrangements approximately 30 % of the time. Thus, in both these cases, our method confirms the current annotation for these gene segments. However, we observed a few notable discrepancies: TCRβ V gene segments TRBV6-8 and TRBV6-9 are currently annotated as functional; however, our analysis suggests the existence of pseudogenic alleles, because they were only found in productive rearrangements approximately 20 % of the time. Moreover, we observed several examples of genes annotated as having only functional genes in IMGT that appear to have both functional and non-functional alleles that segregate in this cohort. Finally, we saw one gene segment annotated as having both functional and non-functional alleles that consistently behaved as a functional gene segment in this cohort, while a second gene segment annotated as having both functional and non-functional allele consistently behaved as a pseudogene in this cohort.

Importantly, the method described in this study, which is ideally suited to study the TCRB locus, has the potential to be applied to other loci that undergo selection such as the α, γ, and δ gene segments of the TCR and the genes that encode for immunoglobulins. Although subtleties related to, for example, the different selective pressures that γ and δ T cells are exposed to will require some modifications to this approach [19], we expect that the basic logic described above will be applicable to other loci. In closing, we hypothesize that population-level variation in the repertoire of available TCR/immunoglobulin gene segments may represent an underappreciated source of heritable diversity in the adaptive immune system.

## Additional file

Additional file 1:
**Table listing the number of sequences observed in the study (including total, unique, productive, and non-productive) that used each V gene segment in each individual.** (XLSX 1183 kb)
